# Review of the Functions of Archimedes’ Spiral Metallic Nanostructures

**DOI:** 10.3390/nano7110405

**Published:** 2017-11-22

**Authors:** Zhongyi Guo, Zixiang Li, Jingran Zhang, Kai Guo, Fei Shen, Qingfeng Zhou, Hongping Zhou

**Affiliations:** School of Computer and Information, Hefei University of Technology, Hefei 230009, China; lizixiang_00@163.com (Z.L.); zhangjingran51@126.com (J.Z.); kai.guo@hfut.edu.cn (K.G.); shenfei@hfut.edu.cn (F.S.); enqfzhou@hfut.com (Q.Z.)

**Keywords:** surface plasmon polaritons, circular polarization (CP), archimedes’ spiral structures, orbital angular momentum (OAM)

## Abstract

Here, we have reviewed some typical plasmonic structures based on Archimedes’ spiral (AS) architectures, which can produce polarization-sensitive focusing phenomenon and generate plasmonic vortices (PVs) carrying controllable orbital angular momentum (OAM) because of the relation between the incident polarized states and the chiralities of the spiral structures. These features can be used to analyze different circular polarization states, which has been one of the rapidly developing researching topics in nanophotonics in recent years. Many investigations demonstrate that the multifunctional spiral-based plasmonic structures are excellent choices for chiral selection and generating the transmitted field with well-defined OAM. The circular polarization extinction ratio, as an evaluation criterion for the polarization selectivity of a designed structure, could be effectively improved by properly modulating the parameters of spiral structures. Such functional spiral plasmonic nanostructures are promising for applications in analyzing circular polarization light, full Stokes vector polarimetric sensors, near-field imaging, and so on.

## 1. Introduction

Polarization, as one of the basic properties of the light, is widely used in modern sensor systems [1], by which the important information of reflecting objects can be obtained and recognized because there are different polarization characteristics for different objects. Additionally, this property can be also employed in other applications, including optical communications [2], imaging polarimetry [3] and microscopy [4]. Investigations of acquiring the linear polarization state have been performed very well from the visible [5,6] to infrared range [7]. In contrast, it is still a challenge to directly detect and analyze the circular polarization state, where a quarter wave plate and a linear polarizer are simultaneously needed. However, the utilization of bulky optical devices hinders the miniaturization of integrated optical system [8,9]. The emerged nano-scale metallic structures, which produce a strongly polarization-dependent optical response, can be a promising alternative for obtaining polarization information.

Such Archimedes’ spiral plasmonic structures can be used as the circular polarization analyzer for replacing a quarter wave plate combined with a linear polarizer, which will offer the possibility for potentially miniature integration. At an early time, the Archimedes’ spiral configurations served as spiral antennas due to the broadband characteristics as circularly-polarized radiators [10,11,12,13], which can also provide a new idea for the underlying polarization-dependent effect. In recent years, more attention has been paid to the Archimedes’ spiral structure with regard to its functionality for circular polarization analysis and generating arbitrary orbital angular momentum (OAM) under illuminations of circular polarization light (CPL). The angular momentum of an optical beam contains the intrinsic component of the spin angular momentum (SAM), associated with the handedness of circular polarizations, and the extrinsic component of the OAM originating from the spiral phase profile of the beam around the axis of propagation. These spiral structures have brought about extensive investigations for optical applications in polarization selection, polarization conversion, plasmonic focusing and selective trapping [14,15,16,17,18,19,20].

In this review, we firstly review several nanostructures based on Archimedes’ spiral, including the pure single-turn Archimedes’ spiral slit (ASS), multiple-turn ASS, a hybrid spiral plasmonic lens, a concentric annular groove surrounded by ASS, Archimedes’ spiral gratings (ASG) combined with ASS, a metal-insulator-metal (MIM) waveguide combined with ASS, the aberrance of ASS, and so on, which can realize the functions of selecting and analyzing the polarization states of CPL incidences. These nanostructures have distinct superiority in miniaturization and analyzing the CPL incidences with high efficiency. In addition, they can generate the optical vortex light carrying well-defined OAM. The construction of double spiral gratings with central hole dynamically switches the incident CPL into the beams with different OAMs due to the extra OAM originating from the spiral geometry structure. The introduction of the symmetric broken Archimedes’ spiral geometry structure supplies the opportunity to nano-scale optical elements for sensing applications and convenient optical integration for measuring full Stokes-vectors of the polarization information.

## 2. The Polarization-Sensitive Mechanism

**Figure 1:**
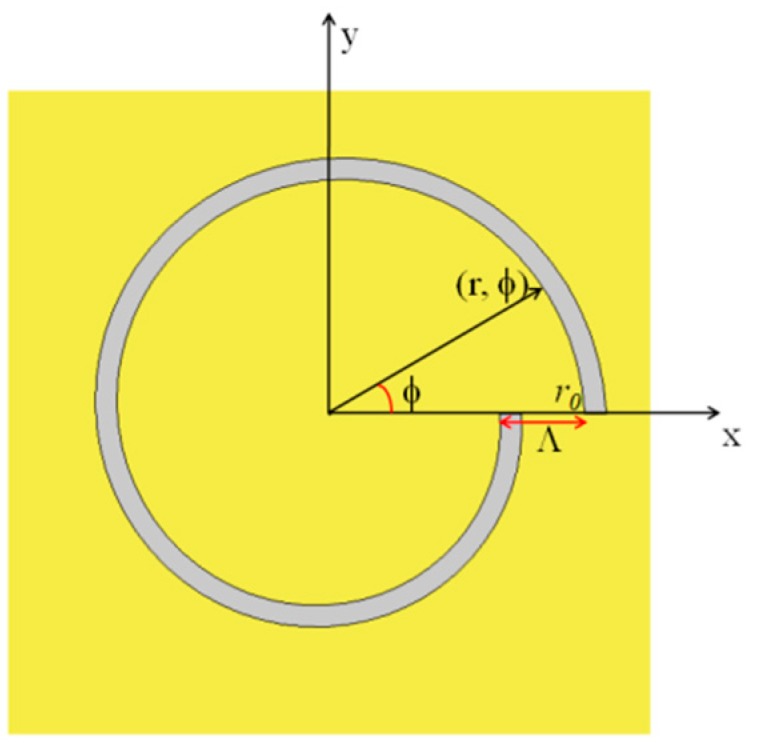
Schematic diagram of a single-turn left-handed Archimedes’ spiral slit (ASS) etched though a thin gold film.

The schematic diagram of a single-turn left-handed ASS in the cylindrical coordinates is illustrated in Figure 1, and its geometry can be described in a simple algebraic form. The geometric design of the ASS can also be called the plasmonic vortex lens (PVL). The distance r from the center of the PVL to the slit with the azimuthal angle *φ* is given by *r = r*_0_ − *Λ × φ/*2π where *r*_0_ is the initial radius of the PVL, and *Λ* is the wavelength of surface plasmon polaritons (SPPs) [21,22].

**Figure 2:**
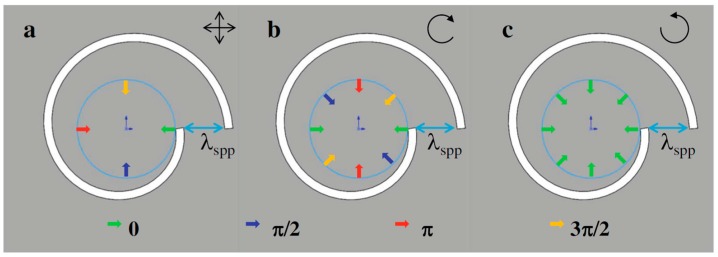
Schematic diagram of the phase of the generated surface plasmon polariton (SPP) wave at the virtual contour with the dashed black line inside the left-handed ASS by: (**a**) radial polarized light; (**b**) left circular polarization (LCP) incidence; the topological charge of the SPP vortex is *j*_pv_ = 2; (**c**) right circular polarization (RCP) incidence; the topological charge of the SPP vortex is *j*_pv_ = 0. Reproduced with permission from [28]. Copyright Springer, 2015.

The mechanisms of polarization-sensitive focusing and generating OAM for the ASS have been exploited adequately [23,24,25,26,27]. In particular, the first study on the chiral surface plasmon induced by the ASS was reported in 2006 [25], in which the chirality of the system is defined by the incident CPL associated with the incident topological charges and the rotating directions of the incident CPL along its propagating directions, as well as the ASS associated with the order of the structural azimuthal variation. The CPL is considered to carry the SAM of *σ = s*_i_ħ, where *s*_i_ = +1 and −1 for the right circular polarization (RCP) and left circular polarization (LCP), respectively, and ħ is Plank’s constant h divided by 2π [26]. The generated plasmonic vortex can be characterized by topological charge *j*_pv_ whose formula [28] can be expressed by:

(1) $$j_{\mathrm{pv}}=m+l_{i}+s_{i}$$

where *m* is a parameter denoting the chirality of the PVL and *s*_i_ and *l*_i_ represent the SAM and OAM of the incident photons. Owing to the geometric asymmetry, such a spiral structure can provide extra phase compensation. The phase compensation mechanisms of the spiral structure under different incidences are illustrated in Figure 2. Figure 2a corresponds to the incident case of radially-polarized light, and Figure 2b,c corresponds to the CPL incidences with different chiralities respectively for the proposed left-handed ASS. When the radially-polarized light or CPL illuminates the spiral plasmonic structure, the entire beam is in transverse magnetic (TM) polarization with respect to the slit wall, enabling SPPs excitation with initial phases from all directions. The colored arrows indicate the propagating directions and the phase difference of the generated SPP waves. As shown in Figure 2a, owing to the non-chirality of the radially-polarized light, the phase compensation is only provided by the geometric phase from the ASS whose chirality is −1. For the CPL incidence, there is no OAM, so *l*_i_ = 0. Actually, the interaction between the incident light and the anisotropic (inhomogeneous) nanostructure is the superposition of the SAM, the OAM and the ASS chirality on the SPPs’ interference. When the CPL carrying proper SAM illuminates the ASS, the ASS can act as a converter from the SAM to OAM [29,30]. When the rotation direction of the spiral structure is the same as that of polarized incident light shown in the Figure 2b (namely, the spiral structure and the incident light have the same chirality), the excited SPP wave carries the phase including the initial phase from the CPL and the geometric phase determined by the radius mismatch. In this case, the SPP waves reach the center of the ASS with opposite phases and interfere destructively, which will form the Bessel beam with a topological charge of *j*_pv_ = 2. While the RCP incident case is given in Figure 2c, the SPP waves that arrive at the center of PVL have the same phase and then interfere constructively, which will form the Bessel beam with a topological charge of *j*_pv_ = 0. Thus, the generated beams for incident light with opposite chirality have different topological charges and obviously different field distributions.

The above-mentioned relationship can be denoted by the angular momentum conservation law and the dominant vertical electric field of the SPP wave at the concentric position of the PVL, which can be simply expressed by the Bessel function as:

(2) $$E_{z}\left(r,\varphi ,z\right)\propto E_{0}\exp(ik_{z}z)\exp(i(s_{i}+m)\varphi )J_{\left(s_{i}+m\right)}(k_{\rho }r)$$

where $k_{\rho }$ and *k_z_* denote the wavenumber radial to the center axis and perpendicular to the metal plate, respectively, (r,φ) is the polar coordinate corresponding to the Cartesian coordinate(*x*,*y*).

In general, the transmitted intensities of the ASS can be simulated by finite-different time-domain (FDTD: FDTD Solutions) or the finite element method (FEM: COMSOL Multiphysics) with the proper boundary conditions [28]. In experiments, the different transmitted intensities’ distributions can be detected by a metallic apertured near-field scanning optical microscope (NSOM) fiber probe system [31].

## 3. Different Structural Constructions

### 3.1. Pure Archimedes’ Spiral Plasmonic Lens

**Figure 3:**
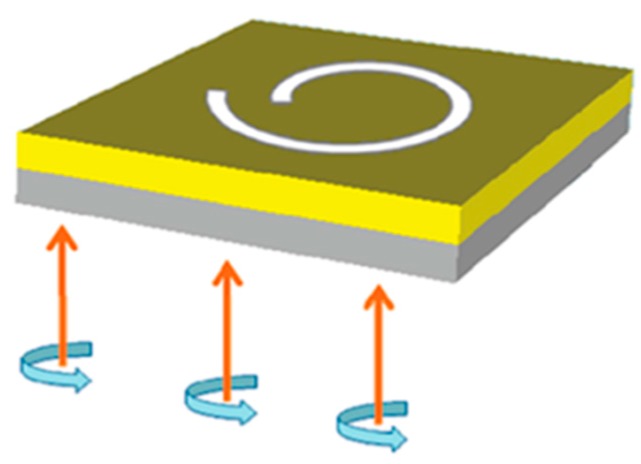
Schematic diagram of a single-turn left-handed Archimedes’ spiral plasmonic structure with CPL illumination. A single-turn left handed spiral (LHS) slit was etched through the gold film, which was deposited on the glass substrate. Reproduced with permission [31]. Copyright American Chemical Society, 2010.

The single-turn Archimedes’ spiral plasmonic lens was designed and investigated as a miniature circular polarization analyzer in 2009 by Q. Zhan [31,32]. The schematic diagram of a single-turn left-handed Archimedes’ spiral plasmonic lens under CPL illumination is illustrated in Figure 3, in which the width of spiral slit is set as 200 nm. The spiral constant of *r*_0_ in the above algebraic formula was set as *r*_0_ = 2*Λ*, and the incident wavelength was chosen to be 808 nm.

Such a spiral plasmonic lens has been investigated analytically and experimentally for its sensitivity to the incident CPL polarizations. With increasing radius of the spiral slit, the structure can generate a phase shift to the propagating SPP wave, which will finally adjust the phase of the SPP wave at the structural center. Hence, such a miniature geometry structure processes the feature of focusing the CPL with a given handedness into a confined spot while defocusing the CPL with the other handedness into a donut-shaped point, which can be used for selecting the polarization states of CPL incidences. Besides, for this type of spiral plasmonic lens, there is no need to adjust the central alignment (axially symmetric structures [33,34,35,36] required to align the center of the circularly-polarized beam and the center of the plasmonic lens), which will broaden the real applications in parallel imaging [37].

**Figure 4:**
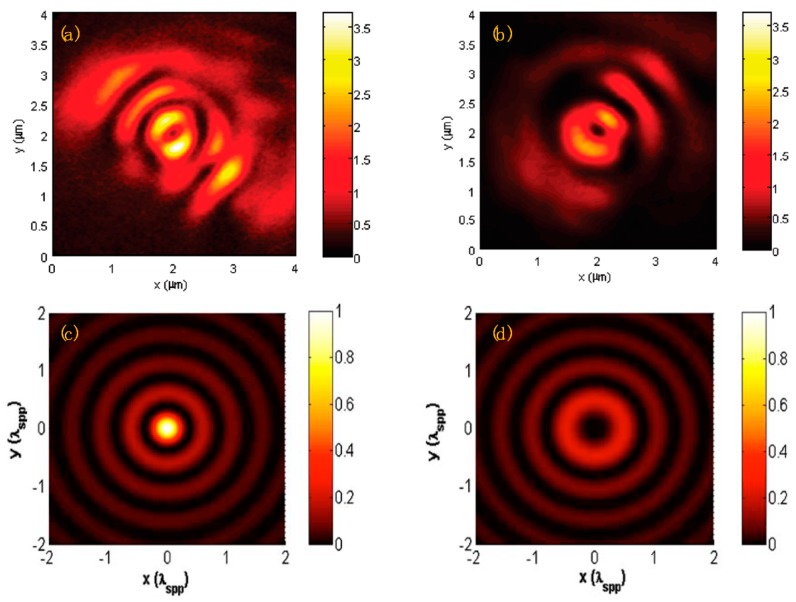
Measured near-field scanning optical microscope (NSOM) images at the air/gold interface (**a**,**b**); Reproduced with permission from [31], Copyright American Chemical Society, 2010. Analytical calculation surface intensity distribution (**c**,**d**); Reproduced with permission from [32], Copyright Optical Society of America, 2009. For an LHS lens structure under (**a**,**c**) RCP illumination and (**b**,**d**) LCP illumination.

The near-field intensity distributions are shown in Figure 4a,b for left-handed ASS under CPL illuminations, which are measured by a metallic apertured near-field scanning optical microscope (NSOM) fiber probe. The measured results agree well with the theoretical analysis as shown in Figure 4c,d, except for a dark center intensity distribution under RCP illumination as shown in Figure 4a because the metallic apertured NSOM fiber probe is more sensitive to transversal components [38,39]. However, such an experimental phenomenon does not contradict a confined spot in theoretical analysis since the peak detected signal of the NSOM fiber probe in the focus region under RCP incidence is still two-times higher than that under LCP light incidence.

Such a symmetry broken structure of the spiral slit lens can be easily fabricated into gold film and selectively focus LCP incidence and RCP incidence into spatially-separated fields. By means of NSOM or two-photon fluorescence microscopy [40], the surface intensity distributions can be detected. The generated SPPs propagating toward the center of the plasmonic lens interfere constructively with the same phase or destructively with the opposite phase, which led to a zeroth-order evanescent field distribution with a central peak or a donut-shaped second-order evanescent field distribution, respectively.

Both the experimental and analytical results of surface intensity distributions under RCP and LCP incidences match well with the simulations [32]. By adjusting the structure parameters of PVL and incident wavelength, the optical field can be focused into the far-field region, which is demonstrated by finite-difference time-domain simulation and experiments [41]. In this work, the *r*_0_ of the single-turn ASS is set to be 2 μm, and the free space wavelength is chosen to be 405 nm. A tightly focused spot with the size of 330 nm nearly at 3.5 μm from the metal surface can be obtained, when the structural chirality is opposite the chirality of the CPL incidence. This focusing enhancement in the far-field region is because of the short wavelength [42,43] and the constructive interference of the SPP wave at the center of the plasmonic lens. These far-field properties make it possible to probe the signal of sample in the far-field, therefore taking the place of conventional near-field optical devices. which suffer from a very short working distance.

### 3.2. Multiple-Turn Spiral Slit

**Figure 5:**
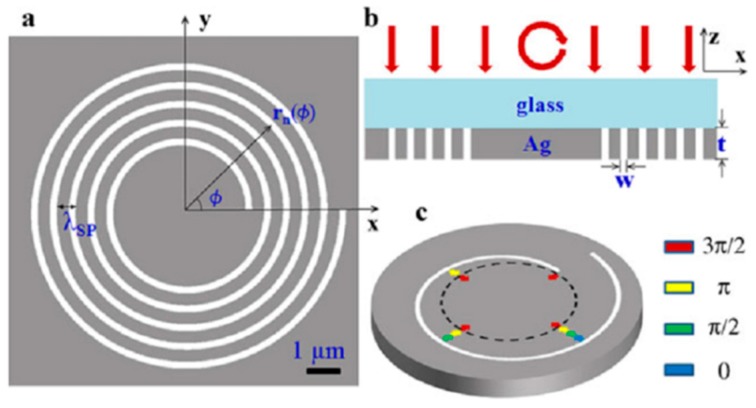
(**a**) Schematic diagram of left-handed multiple-turn plasmonic lens; (**b**) RCP light at a wavelength of 660 nm illuminates along the negative *z*-coordinate (**c**) schematic diagram of the relative phase of surface plasmon waves excited by the spiral slit under RCP illumination. The different color arrows represent the different phases’ increment of the electric field *E_z_*. Reproduced with permission from [44]. Copyright Springer, 2011.

After the single turn spiral slit was investigated, the performance of multiple-turn ASS shown in Figure 5 was reported in 2011 [44]. Based on the same phase matching theory, such a multiple-turn spiral structure works in a more effective and practical way for the application of plasmonic lens because the increasing turns will improve the coupling effects of the generated SPP wave.

The schematic diagram of the left-handed five-turn spiral slit etched through 300-nm silver film as a plasmonic lens is shown in Figure 5a. The RCP illuminates along the negative *z*-coordinate on the structure with the wavelength of 660 nm as depicted in Figure 5b. Figure 5c illustrates the phase retardations of the generated SPP wave along the propagation direction with colorful arrows, which means that the excited SPP wave carrying the initial phase at the ASS experiences phase retardations proportional to the distance from the slit to the center when it propagates from the slit to the center of the ASS, as we analyze above. Additionally, they will keep the same phase once they reach the center of the ASS and finally lead to a constructive interference pattern.

The FDTD simulation results for such multiple-turn ASS demonstrate its advantages for generating a high quality, zero-order evanescent Bessel beam and relative high focusing depth of the focus spot under RCP illumination. While under the LCP incidence, a dark center will be obtained, as expected. Thus, such multiple-turn ASS will be quite useful in detecting the polarization characters of the incident CPL. Nevertheless, a fact should be noted that the intensity will reach the maximum when the turns are increased to be five owing to the limitation of the propagating length of the SPP wave. Such a multiple-turn ASS can find potential applications for focusing beams, intensity actuating, CPL polarization analysis, and so on.

### 3.3. Hybrid Spiral Plasmonic Lens

**Figure 6:**
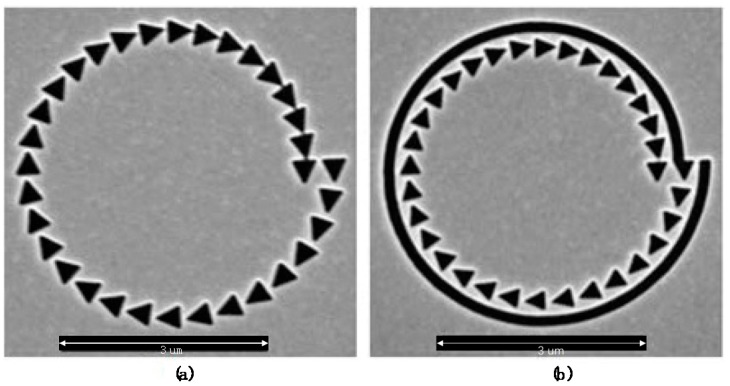
Scanning electron microscope(SEM) image of a plasmonic lens that consists of a spirally-arranged triangular sub-aperture array etched into a gold film (**a**) and a hybrid spiral plasmonic lens combining the array of triangular apertures with single-turn ASS (**b**) fabricated with focused ion beam (FIB) milling. Reproduced with permission from [46]. Copyright Optical Society of America, 2012.

**Figure 7:**
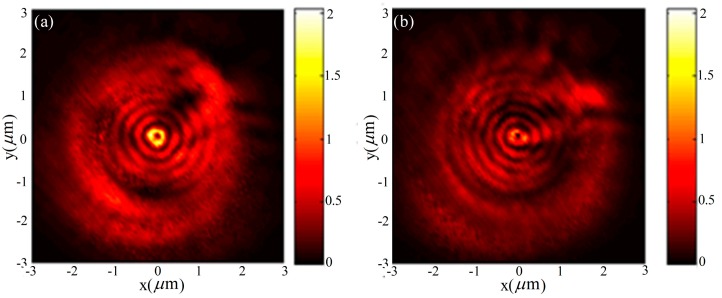
Measured NSOM near-field intensity distribution images of the left-handed arranged spiral triangular aperture array under (**a**) RCP and (**b**) LCP incidence. Reproduced with permission from [46]. Copyright Optical Society of America, 2012.

The ASS mentioned above can only couple the radially-polarized component of circularly-polarized incidence into SPP waves. On the base of the single-turn ASS, a hybrid spiral plasmonic lens has been further proposed for enhancing the coupling efficiency [45,46] (as shown in Figure 6). It combines a single-turn ASS with a single-turn arranged triangular aperture array, coupling both the radial and the azimuthal polarization components of CPL. The experimental and numerical results verify the expectations of the design very well. As shown in Figure 6a, the spiral arrangement of 32 isosceles triangular apertures etched into a 150-nm gold film can couple both the radial and the azimuthal polarization components of CPL [47]. When the azimuthal component is incident to the triangular apertures’ array, constructive focusing can be achieved owing to the in-phase surface plasmons at the two equal sides and adjacent aperture. When the radial component is incident to the triangular aperture array, destructive focusing will be achieved owing to the out-of-phase surface plasmons at the two equal sides and adjacent aperture. Such a left-handed spiral triangle lens can focus the RCP and LCP incidence into different patterns of the electric field distributions. The measured intensity distributions by NSOM for the left-handed spiral triangle lens under RCP and LCP illuminations are shown in Figure 7a,b, respectively. Besides, the spiral arrangement of triangular array (symmetry broken) does not need the central alignment of the plasmonic lens to the singularity point of illumination either, which can overcome the difficulty of using the scanning mechanism for imaging.

The distance between the spiral triangular aperture array and the spiral slit is set as 0.75 *λ*_spp_, resulting in a phase difference of 3π/2. The radial and azimuthal polarization components of CPL has a π/2 phase difference. Therefore, an overall 2π phase difference may be obtained between SPP waves excited at the spiral triangular aperture array and the spiral slit, leading to constructive interference at the center with the highest field enhancement. Thus, the combination of the spiral slit with spirally-arranged triangular aperture array shown in Figure 6b can focus RCP light into a more tightly focused spot and defocus the LCP light into a donut-shaped distribution with a dark center. The superiorities of the combination lie in the higher power conversion efficiency and the field enhancement compared with the formerly-designed pure ASS plasmonic lens. The field enhancement at the focusing and the power conversion efficiency of the circular polarization analyzer are increased by 39.53% and 94.69% by numerical studies, respectively.

In terms of the hybrid plasmonic lens, a zeroth-order (second-order) evanescent Bessel vortex beam will be obtained under RCP (LCP) incident illumination with higher coupling efficiency. For both circularly-polarized illuminations, donut-shaped patterns with a dark center are obtained in experimental results owing to the low coupling efficiency of the longitudinal field, while the detected peak signal in the focus region for RCP incidence is obviously higher than that with LCP incidence. One of the methods to improve the coupling efficiency of the longitudinal field is to use the worn-out NSOM fiber probe with a 200-nm aperture. Therefore, a detector with diameter of d placed in the focus region can detect the signals for distinguishing the RCP and LCP well. Additionally, the circularly-polarized extinction ratio better than 16 can be attained with a diameter of the aperture as 0.3*λ*, which shows that it can be a high-efficiency miniature circular polarization analyzer. Besides, an array of these hybrid plasmonic lenses can be also used in parallel near-field and polarimetric imaging.

### 3.4. ASS with a Concentric Annular Groove

**Figure 8:**
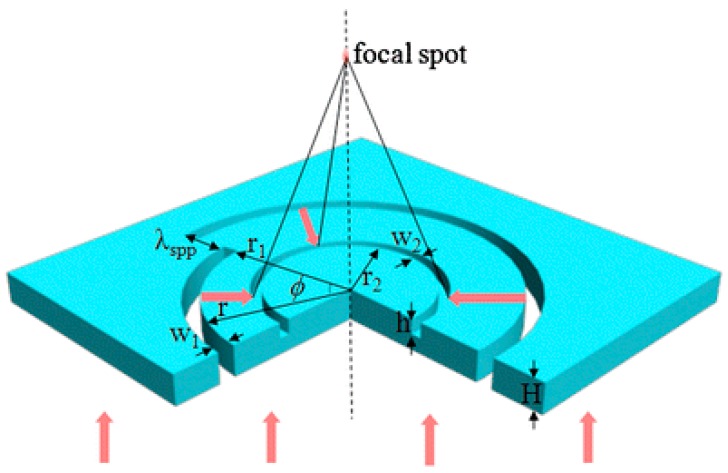
Schematic diagram of the designed structure under the illumination of the RCP plane wave along the positive *z*-direction. Reproduced with permission from [48]. Copyright Springer, 2011.

The combination of ASS and a concentric annular groove, as shown in Figure 8, has also been proposed and investigated in theory for realizing the selection for CPL in the far-field [48]. The improved performance compared with the pure ASS comes from the fact that the existence of the circular annular groove can scatter the SPPs at the groove into the propagating wave and then converge the propagating waves into the far-field. The scattered light by the annular groove can interfere constructively or destructively in the far-field, which make it available and convenient for practical applications in the far-field, where the signal can be detected by conventional optical devices. Simulated results indicate that such a simplified structure can be also used to focus the incident CPL into the far-field region effectively, if the rotation direction of polarization light is opposite the chirality of the ASS. Additionally, the full width at half maximum (FWHM) of the focal spot can always maintain less than the half wavelength of incident light, which can overcome the shortcoming of the near-field characteristics of the intensity distribution.

The schematic diagram (Figure 8) shows a left-handed ASS milled into a 300-nm silver film with a concentric annular groove as a plasmonic lens, and the corresponding parameters are also indicated. The circularly-polarized plane wave illuminates along the positive *z*-direction with wavelength of 660 nm, and the ASS width *w*_1_ and the groove depth *h* are set as 100 and 50 nm, respectively. When the RCP light is incident on the plasmonic lens structure, SPP waves excited by the ASS will propagate along the metal surface, and then, they are scattered by the concentric annular groove into propagating waves into free space. Eventually, the scattered propagating waves from the annular groove will interfere constructively because of the same phase on the *z*-axis.

**Figure 9:**
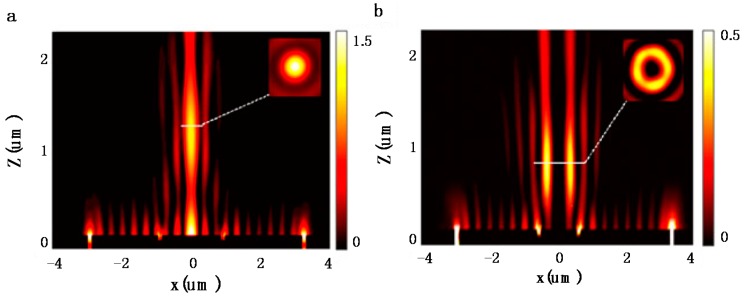
The simulated |*E*|^2^ distributions in the *x*-*z*-plane for the left-hand spiral plasmonic lens under RCP incidence (**a**) and LCP incidence (**b**). Reproduced with permission from [48]. Copyright Springer, 2011.

Figure 9 shows the simulated |*E*|^2^ distribution in the *x*-*z*-plane for the left-handed spiral plasmonic lens shown in Figure 8 with concentric annular groove radius *r*_2_ = 0.90 μm under RCP and LCP incidence, respectively. Such a left-handed combined structure focuses RCP light into a central spot efficiently, as shown in Figure 9a. Owing to the existing scattering phase function of the scattered light caused by the concentric annular groove, the intensity along the propagation direction is enhanced gradually (there is an obvious difference in intensity point by point) into the far-field. The FWHM of the focus is calculated to be 0.44 *λ*_0_ smaller than the half wavelength of incident light (*λ*_0_); while such a structure focuses LCP incident light into a ring with a dark center spot as shown in Figure 9b, due to the destructive interference of scattered light of the SPP wave in the far-field.

The focus effect of similar combined structures with five concentric annular grooves *R* = 0.58, 0.90, 1.22, 1.54 and 1.86 μm are also investigated. Simulated results reveal that the focus depth can be modulated by adjusting the number and radius of concentric annular grooves. The demonstrated potentials make this structure have great applications in the areas of optical detection, imaging, and so on. This plasmonic lens provides new trains of thought for later studies on combing a multiple-turn spiral slit with several grooves, which can improve the coupling efficiency and elongate the focal depth effectively.

### 3.5. ASS Combined with Coaxial Archimedes’ Spiral Gratings 

**Figure 10:**
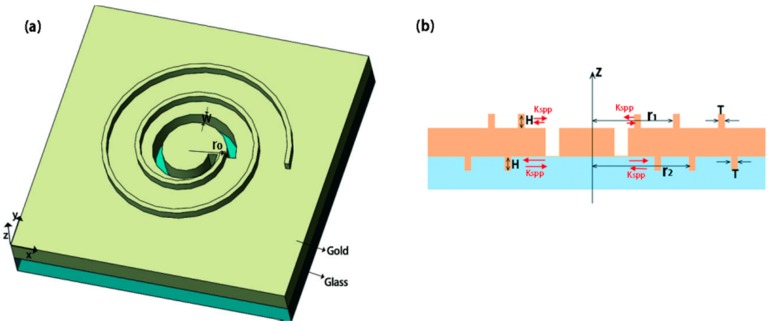
Schematic diagram of the proposed structure under the illumination of the circular polarization plane wave along the positive z-direction (from glass side); (**a**) a single-turn left-hand spiral slit surrounded by Archimedes’ spiral gold grating on both sides of the metal film; (**b**) lateral view of the structure along the location of the line depicted in (**a**). The red arrows represent the propagation of SPPs. The incident light is coupled into SPPs through the spiral slit, and the SPPs generated from the corresponding slits at each side have the opposite direction of propagation. The spiral gratings reflect part of the SPP wave due to Bragg reflection. Reproduced with permission from [28]. Copyright Springer, 2015.

A combined plasmonic structure by ASS with coaxial Archimedes’ spiral gratings (ASG) on both sides of the gold film (Figure 10) has also been proposed and investigated in theory by our group, which is designed based on the same spin-dependent polarization-sensitive and spin-dependent focusing effect of the ASS [28]. The designed structure is sensitive to incident polarization of CPL, and it can realize the focusing and defocusing effects under the illumination of different CPL effectively owing to the existence of ASG.

A combined plasmonic structure by ASS with coaxial Archimedes’ spiral gratings (ASG) on both sides of the gold film (Figure 10) has also been proposed and investigated fully, which is designed based on the same spin-dependent polarization-sensitive and spin-dependent focusing effect of the ASS [28]. The designed structure is sensitive to incident polarization of CPL, and it can realize the focusing and defocusing effects under the illumination of different CPL effectively owing to the existence of ASG.

The additional ASG structure makes more incident light coupled into SPPs for enhancing the field intensity [49]. The incident light is propagating from the glass side to the metal ASS side. By adding the Archimedes’ spiral gratings on the bottom of the metal, the coupling efficiency of incident light to SPPs could be enhanced efficiently. Meanwhile, with the method of adding ASG on the top of the metal film, the exocentric SPPs can be diminished, and it can decouple into scattering light; therefore, the focus intensity will be stronger.

**Figure 11:**
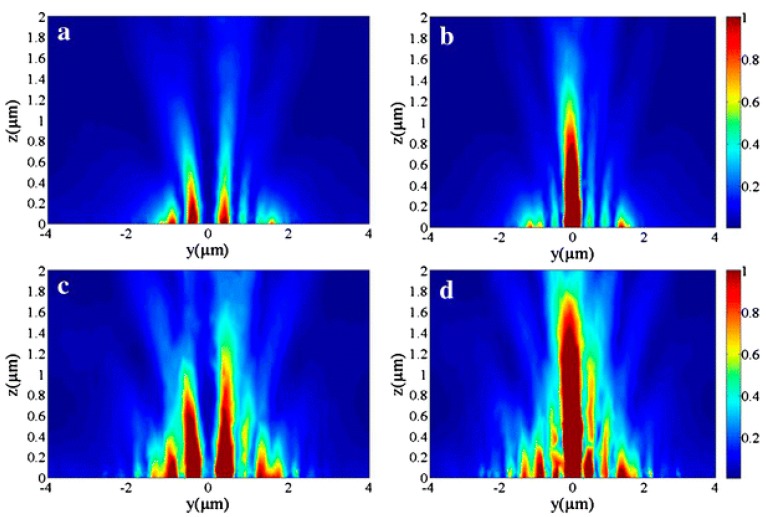
|*E*|^2^ distribution in the *z*-*y*-plane for the left-hand spiral plasmonic lens. Top column (**a**,**b**): Pure single-turn left-hand ASS; bottom column (**c**,**d**): left-hand ASS surrounded by left-hand Archimedes’ spiral grating (ASG) on both sides of the metal film; (**a**,**c**) LCP illumination; (**b**,**d**) RCP illumination. Reproduced with permission from [28]. Copyright Springer, 2015.

The simulation results are shown in Figure 11. Both the electric field intensity for the LCP and RCP illumination cases are improved obviously compared to that of the single ASS, which is better for practical utility.

### 3.6. Combination of ASG and MIM Waveguide

**Figure 12:**
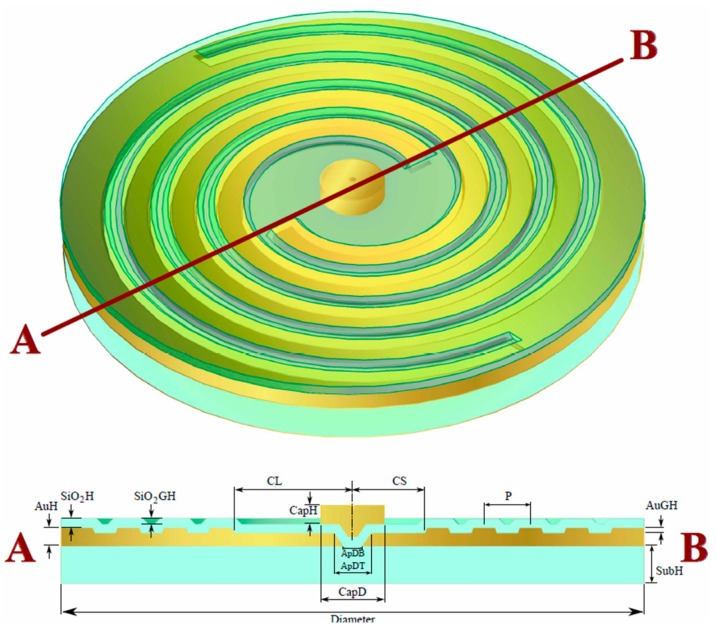
The perspective and cross-section view of ASG with the MIM waveguide. The dimensions of such a structure can be easily fabricated with state-of-the art photolithography systems. Reproduced with permission from [50]. Copyright Optical Society of America, 2011.

The above reviewed structures only work at a specific wavelength and allow both RCP and LCP light transmit though the plasmonics lens. Therefore, if a structure can work in a relative broadband and block CPL with a given handedness, the performance of polarization selectivity will be better. In 2012, R. Hollingsworth et al. proposed a hybrid structure combining ASG with an MIM waveguide [50] as shown in Figure 12. In their investigations, the structure can work very well in visible and near-infrared regions or longer wavelengths. Both numerical simulations and experiments have been performed to demonstrate its admirable performance of circularly dichroism.

The combined structure can be fabricated with the state-of-the-art photolithography systems, and only standard thin film of three lithography levels is required for the fabrication process. The gold film was deposited on the silicon dioxide (SiO_2_), and the center region of the metal spiral gratings was cut out to form a cavity with an aperture whose cross-section was a trapezoid MIM waveguide [51,52,53]. The dielectric layer of SiO_2_ uniformly covered the metal region, and a metal cap was used to cover the circular aperture as shown in Figure 12. The ASG is similar to longer wavelength antenna analogues [10], which can only transmit CPL with a specific chirality, but block CPL with the opposite chirality. Meanwhile, the metal cap can effectively obstruct the direct transmission of the incident light. Such a spiral configuration can concentrate the RCP light and then radiate the energy out of the circular aperture into the far-field region. However, for the LCP light, the field is concentrated into the plasmonic vortex, which embraces the metal cap, and finally, the energy will be absorbed owing to the ohmic losses. These facts indicate that the novel model is a kind of transmitting structure, and the polarization character of CPL incidence can be detected by measuring the far-field transmission. The superiority of this designed structure makes it easy to detect the signals by measuring the far-field transmission and to modulate the concrete operating wavelength. By varying the incident wavelength, the transmission for RCP incidence is still larger than that under LCP illumination in a relatively broad operating wavelength. This is why this structure can work in a controllable operating wavelength. Meanwhile, by modulating the parameters (spiral arm length), more incident energy can be coupled into the SPP mode, and the total transmission can be increased correspondingly under RCP incidence.

**Figure 13:**
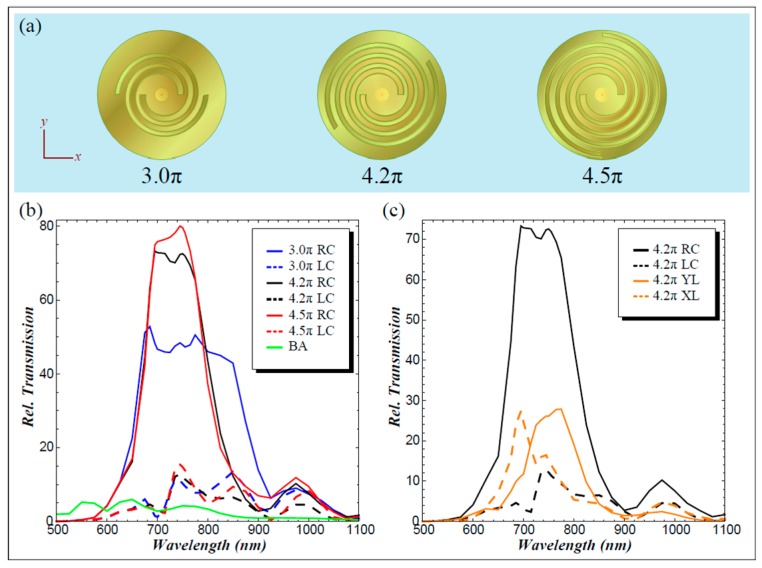
Simulated far-field transmission results. (**a**) Top view of the modeled geometries with different spiral arm lengths; (**b**,**c**) the relative transmissions normalized to the power incident on the circular aperture: (**b**) investigation of the spiral arm lengths; (**c**) comparisons among the RCP, LCP and linear (*x* and *y*-directions) incidences. Reproduced with permission from [50]. Copyright Optical Society of America, 2011.

The investigation on the influencing performances of the arm length (from 3π to 4.5π) are shown in Figure 13. The simulation results reveal that by increasing the arm lengths, the RCP transmission increases, while LCP transmission nearly remains constant for a relatively broad operating wavelength, which means circular polarization selectivity can be improved with increasing the arm lengths. The most representative parameter for evaluating the actual performance of the designed structures is the extinction ratios. The extinction ratios can reach their maximum when the arm length goes to 4.2π, and then, it will slightly reduce with the increased arm length because of the slightly increasing LCP transmission. The simulated results agree well with the measured far-field transmission, which demonstrates that such a configuration can be utilized individually as a miniature circular polarization analyzer with a controllable bandwidth.

### 3.7. The Derivative ASS Structure

**Figure 14:**
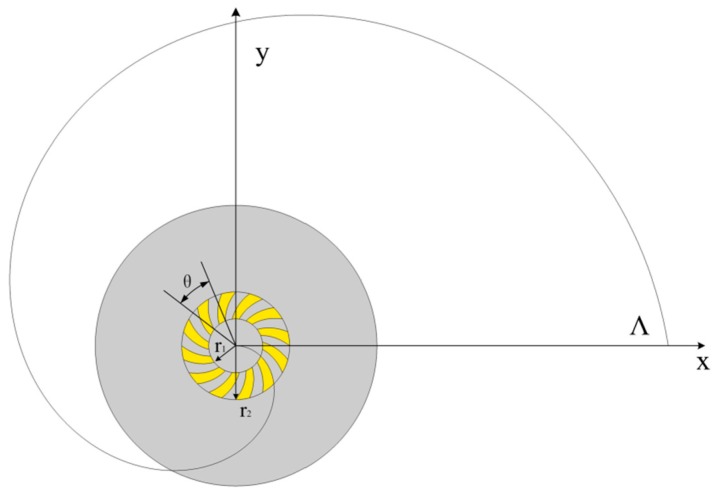
Schematic diagram of the metallic spiral-ring structure. Circularly-polarized illumination (620 nm) is along the *z*-direction, which comes out of the plane. The metallic spiral ring with a 200-nm inner radius *r*_1_ and a 400-nm outer radius *r*_2_ was etched through a 200-nm gold film deposited on the silica substrate. Reproduced with permission from [54]. Copyright Society of Photo-Optical Instrumentation Engineer, 2014.

With the development of the ASS structures, Bo Yan et al. presented a new aberrance based on the algebraic formula of simple ASS [54]. The chiral metallic spiral-ring structure contains discrete metallic slits with curved edges whose top view is depicted in Figure 14. In Cartesian coordinates, the spiral’s slit can be described as follows:

(3) $$\left(\begin{array}{l} x_{i}\\ y_{i} \end{array}\right)={\left(\begin{array}{cc} \cos(\theta /2) & -\sin(\theta /2)\\ \sin(\theta /2) & \cos(\theta /2) \end{array}\right)}^{i-1}\left(\begin{array}{l} \Lambda (1-\varphi /2\pi )\cos(\varphi )\\ \Lambda (1-\varphi /2\pi )\sin(\varphi ) \end{array}\right),i=1,2...4\pi /\theta$$

**Figure 15:**
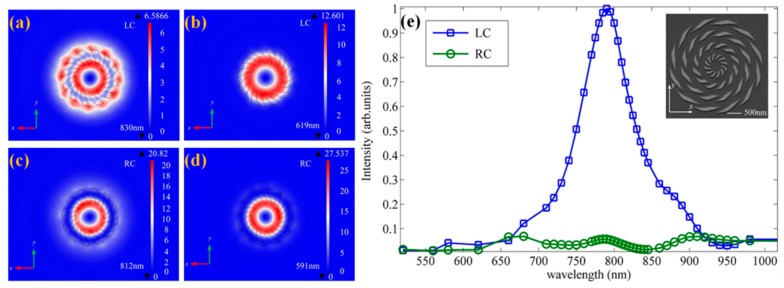
(**a**–**d**) The electric fields’ distributions of *E_z_* by spiral-ring structures under LCP and RCP illumination at the peak intensity wavelength; (**e**) relative transmission spectra of a metallic spiral four-ring structure under LCP and RCP illumination, respectively. Inset: the image of the metallic spiral four-ring structure. The field intensity is calculated through an aperture with a radius of 200 nm, which is located 100 nm from the gold surface. Reproduced with permission from [54]. Copyright Society of Photo-Optical Instrumentation Engineer, 2014.

This new spiral ring structure is a type of ASS aberrance according to Equation (2). The entirety of the structure is a circular shape. The spiral slit is along the Archimedes’ spiral route, and the adjacent spiral route is rotated by 60°, which is small enough for exciting the localized surface plasmons (LSPs) [55] between the adjacent metallic slits. LSPs will contribute to extraordinary light enhancement in nano-scale metallic parts, which are non-propagation excitations and do not require the phase-matching conditions [56,57]. The inner radius *r*_1_ and the outer radius *r*_2_ are set to be 200 and 400 nm. The scattering characteristics for one metallic part are primarily studied at the near-field region (just 100 nm above the gold surface) at a 620-nm operating wavelength for RCP and LCP illumination. As a result, the energy is concentrated into different regions for opposite CPL incidence owing to the spin-dependent effect. Additionally, the obvious differences in the intensity distribution of the *E_z_* component are caused by the different phase delays of LSPs, indicating its potential utilization in modulating the near-field distribution for the opposite CPL incidences. By calculating the total electric field intensity through a specific aperture under illumination, the localized modes have been investigated to observe the resonant peak for LCP and RCP cases, respectively. The obtained resonant peaks for LCP are at 619 and 830 nm, shown in Figure 15a,b, respectively, and those for the RCP case at 591 and 812 nm are shown in Figure 15c,d, respectively. This fact indicates that the localized plasmon resonance due to the curved edges of the metallic slit occurs at different positions (inner/outer radius) for the shorter/longer incident wavelengths, respectively. The excited LSP modes along the circular have the phase delay resulting from the spiral phase; thus, phase distributions by spiral ring plasmonic structures are opposite under different CPL illuminations.

The spiral four-ring structure (shown in the inset of Figure 15e) manufactured by focused ion beam (FIB) in a 200 nm-thick gold film was further investigated. Additionally, the experimental results of the transmission spectra referring to the normalized intensity were detected through an aperture with a radius of 200 nm, about 100 nm above the gold surface, as depicted in Figure 15e. The experimental results show that such a spiral four-ring plasmonic structure can effectively allow the LCP light to penetrate the structure while blocking the RCP incidence absolutely owing to stronger ring-to-ring coupling compared with the spiral single-ring structure in a relatively broadband.

### 3.8. Double Spiral Gratings with a Central Hole

**Figure 16:**
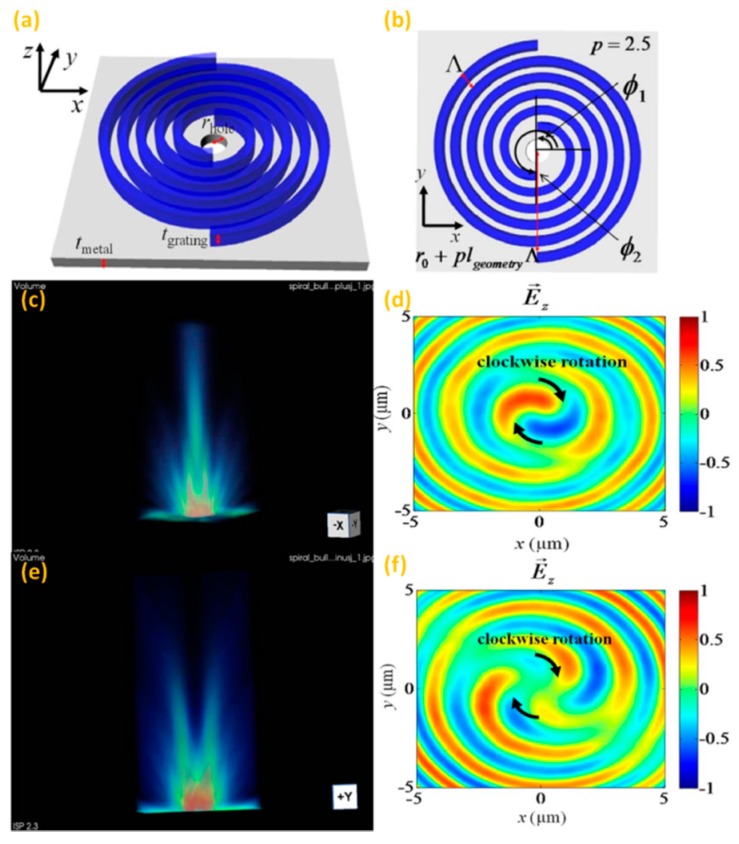
(**a**,**b**) Schematic diagram of the proposed double-spiral configuration with a central hole used for numerical calculation; 3-dimensional (3D) view of the electric field intensity distribution of such a structure with LCP light incidence (**c**) and RCP light incidence (**e**) ranging from 0 to 20 μm above the dielectric grating layer and (**d**,**f**) the vertical electric field (*E_z_*) distribution on the corresponding *z* = 10-μm plane (**c**,**e**) respectively. Reproduced with permission from [58]. Copyright Optical Society of America, 2011.

Based on the polarization-dependent characteristics, these Archimedes’ spiral structures are utilized to generate light possessing a well-defined topological charge. Lee et al. have proposed a kind of plasmonic beaming structure by using the double spiral grating architecture for dynamic switching of the plasmonic beam [58,59,60]. Commonly, plasmonic spiral structures to generate optical beaming are in stark contrast to the plasmonic lens; that is, the transverse field (*E_x_,E_y_*) dominates the electric field component. Such periodic grating structures are designed to efficiently convert the near-field SPPs to the far-field radiations by compensating the momentum mismatch between the SPP mode and the radiation mode, which was firstly demonstrated by using the “bull’s eye” structure for generating plasmonic beaming [61]. The formula of this spiral grating, which similar to the algebraic formula of Archimedes’ spiral, is indicated below:

(4) $$\begin{array}{l} r_{\mathrm{spiral},i(\phi )}=r_{0}+\frac{l_{\mathrm{geometry}}\Lambda }{2\pi }(2\pi p+\phi_{i}-\phi )\\ (i=1,2...n,\phi_{i}\leq \phi \leq \phi_{i}+2\pi p), \end{array}$$

where *r*_0_, *n*, *p* represent the initial radius of the spiral grating, the total number of spiral gratings and the winding number of each spiral, respectively. The subtle difference between Equation (3) and the algebraic formula of Archimedes’ spiral is that in Equation (3), the initial azimuthal angle of each spiral grating is arbitrarily determined by parameter *φ*_i_. The radius mismatch of the structure compensates additional propagation phase delay to the SPP mode, and the additional angular momentum provided by the geometrical structure is called the geometrical angular momentum (*l*_geometry_). The schematic diagram of the proposed structure with a double spiral is shown in Figure 16a,b, where *l*_geometry_ = 2, *r*_0_ = *Λ*/2 + *r*_hole_, *φ*_1_ = π/2, *φ*_2_ = 3π/2, *p* = 2.5, *n* = 2. The advantage of the double spiral over the single one is that the double spiral structure can maintain on-axis beaming characteristics very well, while the symmetry-broken construction of the single spiral will produce distortion of the generated beam. The *l*_geometry_ of the proposed clockwise double-spiral plasmonic structure is two, and the final topological charge of the generated beam is determined by the polarization of incident light. Thus, the generated beams for different CPL incidences have different topological charges and obviously distinct intensity distributions. Figure 16c,e shows the 3D electric field intensity distributions in the range of 0~20 μm above the dielectric grating layer under LCP and RCP incidences, respectively. Figure 16d,f shows the *E_z_* distributions on the *z* = 10-μm plane in two cases correspondingly. The clockwise direction of one cycle detected of the phase modulation near the *z*-axis shown in Figure 16d is determined by the geometrical AM and SAM of incident light (*l*_total_ = *l*_geometry_ + *l*_spin_ = 1), which is opposite the rotation direction of LCP illumination. This result demonstrates the switchable characteristic of the designed structure by changing the polarization states.

When the incident polarization is the same as the rotation of the spiral geometry, the vector sum of tangential field components has a cancellation effect on the axis; therefore, the on-axis beaming generation will be prohibited. The total topological charge is *l*_total_ = 3, which results in a different beam pattern, as shown in Figure 16e, and the generated beam has a dark central intensity distribution. Thus, it is clear that a bright spot can be formed only under LCP illumination, which means such an ASG structure can also be used to select the polarization of CPL. The performance of such a double switchable plasmonic structure can be characterized by the on-off ratio, which is calculated by integrating the time averaged power flow along the *z*-direction passing through a circular region with a concrete diameter (976 nm) situated at the suitable plane (*z* = 8.5 μm) under LCP incidence and then dividing it by that under the RCP case. In this design, the on-off ratio can be obtained as 23.3 dB. Such a double-spiral architecture realizing switchable beam characteristics can find many useful applications in plasmonic data processing, optical sensing and nanoscale optical antennas [62].

### 3.9. Spiral Structure Based on a Nano-Pinhole Array

**Figure 17:**
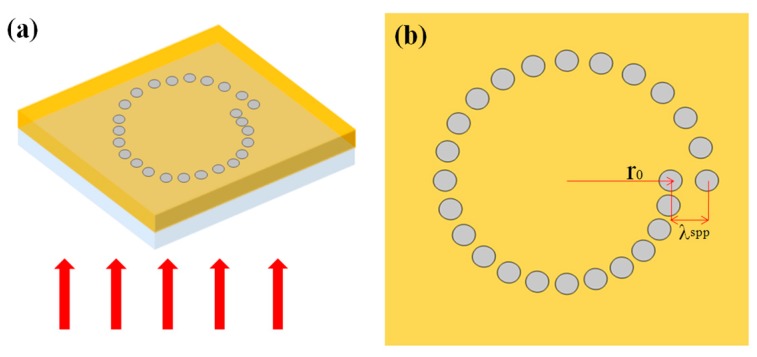
The schematic diagram of the single-turn Archimedean pinholes array (25 pinholes): lateral view (**a**) and the top view (**b**). Reproduced with permission from [63]. Copyright Optical Society of America, 2015.

Recently, we have also proposed a broadband circular polarization analyzer based on a single-turn Archimedean nano-pinhole array [63]. The schematic diagram of the proposed single-turn Archimedean pinhole structure under the incidences of CPL, in which there is a layer of 250-nm gold film deposited on the surface of the glass substrate, are illustrated in Figure 17. The 25 nano-pinholes are arranged along the Archimedean spiral formula of *r = r*_0_ *+ λ*_spp_ *× φ/*2π, which can be etched into gold film very easily in experiments with FIB milling. From the theoretical analysis, the peculiarity of the nano-pinhole array-based structure lies in superposing light fields generated from each dipole sources, which results in enhanced total transmitted longitudinal electric field. Such a designed structure has obviously different electric fields with relatively high extinction ratios at different detecting locations. Besides, it can work at a relative broad operating wavelength from 650 to 900 nm.

**Figure 18:**
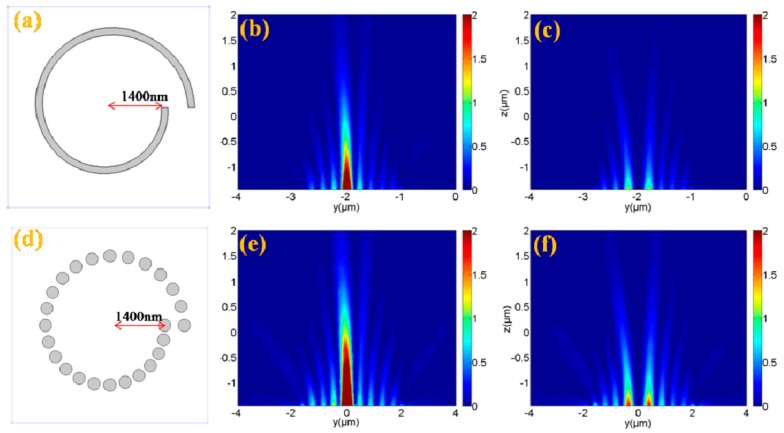
The longitudinal electric field intensity distributions at the *yoz* plane under circular polarization illuminations. (**a**–**c**) for the single-turn Archimedean spiral slit; (**d**–**f**) for the 25 Archimedean nano-pinhole array with *r*_0_ = 1400 nm. Reproduced with permission from [63]. Copyright Optical Society of America, 2015.

The comparisons of the performances between the single-turn ASS with initial radius *r*_0_ *=* 1400 nm shown in Figure 18a-c and the proposed structure (Archimedean nano-pinholes array) with *r*_0_ *=* 1400 nm in Figure 18d-f have been given in the *yoz* plane views under the RCP (middle column) and LCP (right column) incidences, respectively, in which the electric field intensity of the single-turn ASS shown in Figure 18b,c is obviously weaker than that of the Archimedean nano-pinhole array shown in Figure 18e,f The simulated results indicate that the designed Archimedean nano-pinhole array is more effective because of the enhanced coupling efficiency of SPPs. The weaker transmitted field intensity of the traditional ASS is due only to the contribution from the radial dipole sources associated with the radial component, while for our proposed Archimedean nano-pinhole array, the enhanced total transmitted field distribution is originating from superposing fields caused by all dipole sources (the radial dipole sources, the coupling between the radial and azimuthal dipole sources). The simulated results show that such a designed left-handed plasmonic lens can focus RCP light into a confined focus spot and defocus the LCP light into a donut-shaped field, which matches well with the theoretical analysis. Thus, it can be used as a circular polarization analyzer with a high extinction ratio.

### 3.10. Bilayer Holey Spiral Plasmonic Lens

**Figure 19:**
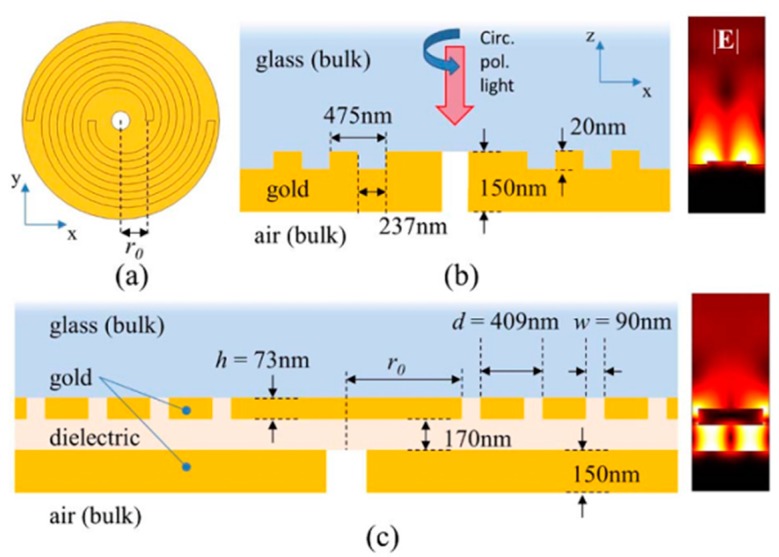
(**a**) Top view and (**b**) cross-section of a single-layer holey plasmonic vortex lens (PVL); (**c**) schematic diagram of the designed holey PVL. The CPL impinges from the glass side with the wavelength of 780 nm. Inserts in (**b**,**c**) are the electric field profiles of the plasmonic modes excited by CPL of two PVLs structures. Reproduced with permission from [64]. Copyright Optical Society of America, 2014.

Pierfrancesco Zilio et al. have also proposed a bilayer holey spiral plasmonic lens as shown in Figure 19 [63]. The transmitting far-field light from the proposed plasmonics lens has been simulated, which demonstrates that the transmitted light carries pure OAM. The top view and cross-section of a single-layer holey PVL are shown in Figure 19a,b. A new PVL with two layers is also illustrated in Figure 19c, and its parameters are all optimized for the working wavelength of 780 nm. Such a PVL with two spirals whose rotated degrees are 360/*m* (here *m* = 2) can improve the coupling efficiency of the PVs, which is the SPPs carrying the angular momentum actually.

The principle of operation is nearly analogous to the single-layer PVL with a circular hole in the center of the lens [65]. For the bilayer architectures, things are obviously different. The existence of the MIM waveguide makes PV in the form of a TM_0_ plasmonic mode [56]. The dielectric layer with a 170-nm thickness as the MIM waveguide only allows the existence of the fundamental TM_0_ plasmonic mode because the thickness is smaller than the cutoff of TM_1_ mode. Thus, the converted PV in the form of an SPP excited by the spiral slit efficiently transmits through the central hole to the air. In contrast, direct transmitted light is blocked. The coupled PV can be fully characterized by topological charge *j*_pv_, as depicted in Equation (1). The transmitted field includes a plasmonic evanescent part with the component of *E_z_* and a propagating spherical wave in the far-field with transverse components *E_x_* and *E_y_*. The topological charge of the propagating spherical wave can be expressed as follows [65]

(5) $$l_{f}=j_{\mathrm{pv}}-\mathrm{sgn}(j_{\mathrm{pv}})=m+l_{i}+s_{i}-\mathrm{sgn}(m+l_{i}+s_{i})$$

**Figure 20:**
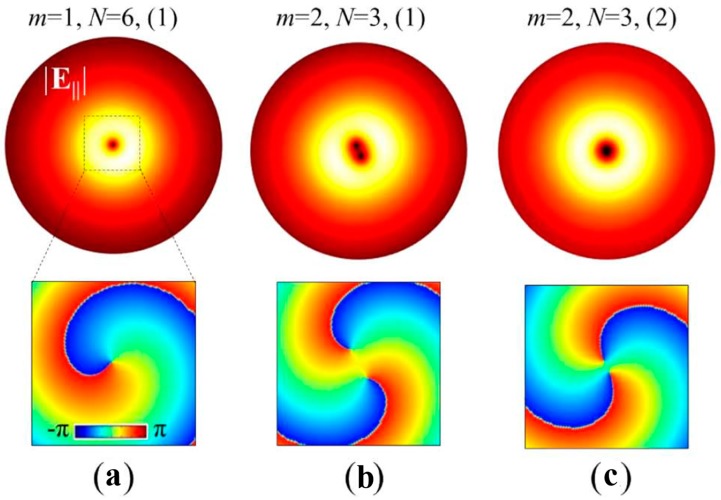
The FEM simulations of the MIM-PVL architecture. The upper column of (**a**–**c**) shows the |*E*_||_| field in the *x*-*y* plane located 800 nm below the hole for the PVLs of *m* = 1, *N* = 6; *m* = 2, *N* = 3; *m* = 2, *N* = 3, respectively. *N* denotes the numbers of spiral turns. The subjacent column displays the corresponding arg(*E_x_*) fields. From [64]; reprinted with permission. Copyright Optical Society of America, 2014.

The simulated results of the electric field and the phase distributions have been demonstrated in Figure 20a–c for three PVLs with specific m and N values. N denotes the number of spiral turns. The |E_||_| field distributions are the cross-section located 800 nm below the hole for the PVL with *m* = 1, *N* = 6, *m* = 2, *N* = 3, *m* = 2, *N* = 3, respectively. The subjacent column displays the corresponding arg(*E_x_*) fields. According to Equation (5), the calculated topological charge *l*_f_ = 1 and 2 of the transmitted *E_x_* are in perfect agreement with the subjacent column of Figure 20. Compared with a single-layer PVL, both the efficiency of transmission and the purity of OAM are improved. Though the entire structure seems more complex than the single layer architecture, only two subsequent FIB millings with the suitable alignment between the two lithographic steps are required for fabrications. As a consequence, such a proposed MIM-PVL architecture can transform CPL illumination into light carrying any specific topological charges in terms of the expected functions. The designed structures will play a significant role in the further investigations of PVs, optical tweezers, optical communication and sensing applications.

## 4. Conclusions

All of above-mentioned configurations take advantage of the polarization-dependent effect resulting from the symmetry-broken character of the Archimedes’ spiral structure, which actually is a chiral metallic structure. Such ASS structures, under illuminations of circular polarization incidences, can effectively excite the SPP wave at the curved slit surface, and then, the generated SPP wave propagates with the initial phase difference determined by the polarization of incident light towards the center. After propagating from the spiral slit to the center of the plasmonic structures, the SPP wave acquires an extra phase delay proportional to the propagation distance and finally interferes constructively or destructively, resulting in the distinct intensity distribution patterns. When the SPP wave from the opposite direction reaches the center with the same phase, a zeroth-order evanescent Bessel beam with a central peak will be obtained. Otherwise, a donut-shaped second-order evanescent Bessel vortex with a dark center will be attained.

For obtaining better SPP coupling efficiency and high power conversion, several additional constructions are proposed to combine with the ASS and multiple-turn AS structures (grooves, gratings and others) for optical sensing applications. A spirally-arranged triangle aperture array is introduced for coupling the azimuthal polarization components of CPL. This hybrid spiral plasmonic lens combining pure single-turn ASS with a spirally-arranged triangle aperture can be used as an efficient miniature circular polarization analyzer for its obvious enhanced field and power conversion efficiency. The combination of the ASS and the coaxial ASGs on both sides of the gold film can improve the efficiency of the incident light coupling into SPPs and diminish the exocentric SPPs contributing to a stronger focus intensity; while the utility of the MIM waveguide at the center of the metal film covered with a metal cap can form a pure transmission structure and such an element can selectively transmit the CPL light with only one given chirality in a broad wavelength. In addition, a novel spiral-ring structure, which contains discrete metallic slits with curved edges, is an aberrance of ASS and concentrates the energy into different regions for opposite circularly-polarized incidence due to the different phase delays for them. Furthermore, a spiral four-ring structure is demonstrated because of its outstanding transmission spectra in a relative broad operating wavelength region for analyzing the polarization characteristics of CPL. Next, the bilayer holey Archimedes’ spiral structure is used as a plasmonic lens due to its function of generating far-field transmission light carrying pure OAM, and double ASGs with a central hole as plasmonic beaming structures are reviewed for their switchable characteristics.

Generally speaking, there are still some other types of Archimedean structures that have been published in recent years, such as rectangular slits array [66,67], a graphene-coated spiral dielectric lens [68,69] and the metallic slit with an auxiliary groove [70], which can function in different applications, such as plasmonic wave manipulation with different propagating directions and plasmonic focusing characteristics. Anyway, by using nano-aperture-based spiral structures, the excited SPP waves can be unidirectionally controlled under different CPL incidences, which provides new paths for manipulating the circular polarization states that can be used as the CPL analyzer and the OAM generator. By combining new shapes of nano-apertures with the proposed spiral structure, the special intensities’ distributions can be obtained in the transmitted fields for special applications in future research. The versatility of Archimedes’ spiral structures makes them conveniently integrated with other subwavelength optical elements and finds more interesting applications in real optics. Meanwhile, this type of ASS structure can be also used in the manipulation of the THz wave, which can be used in THz communication systems, THz imaging systems, and so on.
